# Retinal nerve fibre layer thinning and corneal nerve loss in patients with Bardet-Biedl syndrome

**DOI:** 10.1186/s12920-023-01739-w

**Published:** 2023-11-23

**Authors:** Aziz Belkadi, Gaurav Thareja, Adnan Khan, Nisha Stephan, Shaza Zaghlool, Anna Halama, Ayeda Abdulsalam Ahmed, Yasmin A. Mohamoud, Joel Malek, Karsten Suhre, Rayaz A. Malik

**Affiliations:** 1 Weill Cornell Medicine-Qatar, Qatar Foundation, Education City, Doha, Qatar; 2 Faculty of Health Sciences, Khyber Medical University, Peshawar, Pakistan

**Keywords:** Bardet-Biedl Syndrome, Corneal confocal Microscopy, Nerves, Optical coherence tomography, Retina

## Abstract

**Background:**

Bardet-Biedl syndrome (BBS) is an autosomal recessive, genetically heterogeneous, pleiotropic disorder caused by variants in genes involved in the function of the primary cilium. We have harnessed genomics to identify BBS and ophthalmic technologies to describe novel features of BBS.

**Case presentation:**

A patient with an unclear diagnosis of syndromic type 2 diabetes mellitus, another affected sibling and unaffected siblings and parents were sequenced using DNA extracted from saliva samples. Corneal confocal microscopy (CCM) and retinal spectral domain optical coherence tomography (SD-OCT) were used to identify novel ophthalmic features in these patients. The two affected individuals had a homozygous variant in *C8orf37* (p.Trp185*). SD-OCT and CCM demonstrated a marked and patchy reduction in the retinal nerve fiber layer thickness and loss of corneal nerve fibers, respectively.

**Conclusion:**

This report highlights the use of ophthalmic imaging to identify novel retinal and corneal abnormalities that extend the phenotype of BBS in a patient with syndromic type 2 diabetes.

**Supplementary Information:**

The online version contains supplementary material available at 10.1186/s12920-023-01739-w.

## Introduction

Bardet-Biedl syndrome (BBS) is a rare autosomal recessive, genetically heterogeneous disorder which has been attributed to variants in 26 genes that encode proteins involved in several key signaling pathways, together with alterations in chaperonins and the intraflagellar transport complex [1]. Basal body dysfunction of ciliated cells leads to a heterogenous phenotype with varying expression of obesity, insulin resistance, diabetes mellitus [2], polydactyly, cognitive impairment, renal anomalies, hypogonadism, and rod-cone or cone-rod dystrophy [3]. However, the onset of clinical manifestations is variable, develops progressively and whilst most people are diagnosed in late childhood or as young adults, in some the diagnosis may be missed.

Reduced visual acuity due to retinal dystrophies is a key feature of BBS [4] and a report using optical coherence tomography (OCT) has previously demonstrated outer/inner segment photoreceptor attenuation, but with increased peripapillary retinal nerve fiber layer thickness in a patient with BBS [5]. A *Bbs8* knockout mouse model has shown photoreceptor dysfunction and defects in the retinal outer segment [6]. With regard to neurological manifestations the main focus has been on cognitive impairment with alterations in perceptual intellectual ability, auditory attentional capacity together with ataxia and poor coordination in patients with BBS. *BBS1* and *BBS4* genes encode proteins near the centrioles of sensory neurons, and *Bbs1*^*−/−*^ and *Bbs4*^*−/−*^ mice have shown alterations in the *TRPV1* thermosensory channel and *STOML3* mechanosensory channel with increased thermal and mechanosensory thresholds [7]. To date there are no clinical studies which have evaluated sensory nerves in patients with BBS.

**Fig. 1:**
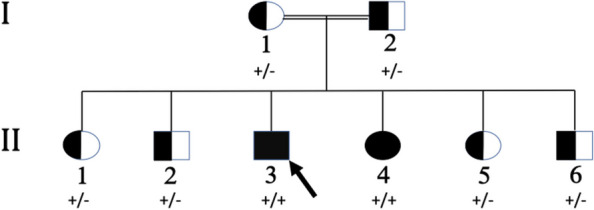
Segregation analysis of the *C8orf37* - stop gain variant p.Trp185* in a consanguineous family with BBS. The proband and affected sister carried the variant in the homozygous state, while the consanguineous parents and unaffected siblings carried the variant in the heterozygous state. Genotypes of the identified C8orf37 variant are indicated by ‘‘+’’ (mutated allele) and ‘‘-’’ (reference allele)

We describe a family of two parents and six siblings with one sibling presenting with a syndromic form of diabetes (Fig. 1). Case report: a 31-year-old male (proband) with obesity, type 2 diabetes mellitus (T2DM), and proteinuria was referred to the endocrinology clinic in Hamad General Hospital, Doha, Qatar in 2012. The proband had impaired vision since the age of 5 years. His sister, aged 33 years (affected sibling) was also obese and had impaired vision at a young age.

We carried out whole-genome sequencing of DNA extracted from the saliva of all family members. A previously reported pathogenic stop gain variant in *C8orf37* - recently renamed “cilia and flagella associated protein 418” *CFAP418 -* (p.Trp185*, rs748014296), known to cause BBS type 21, was found in the homozygous state in both affected individuals, and in the heterozygous state in the parents and unaffected siblings. To further investigate the impaired vision, we used spectral domain OCT (SD-OCT) to quantify the retinal nerve fiber layer thickness [8] and corneal confocal microscopy (CCM) to characterize corneal nerve morphology [9].

## Materials and methods

### Sample collection

Saliva samples were collected from the two patients and their unaffected parents and four siblings, using an Oragene DISCOVER (OGR-500) collection kit (DNA Genotek, Canada) according to the manufacturer’s instructions. 2 mL of saliva was collected into a saliva collecting tube and stirred by inversion to ensure mixing of saliva with the stabilizing reagent and stored at room temperature (RT) for two weeks until DNA extraction. Clinical and demographic parameters, blood pressure, HbA_1c_, total cholesterol, HDL, LDL, and triglycerides were assessed using standard clinical protocols.

### Sample collection, DNA extraction, and quantification

DNA extraction was performed using prepIT.L2P reagent (DNA Genotek, Canada) according to the manufacturer’s instructions. The samples collected in Oragene DISCOVER (OGR-500) collection tubes were incubated at 50 °C in a water bath for 1 h. After incubation, 4 mL of the sample was transferred into a fresh falcon tube, mixed with 160 µL of prepIT.L2P buffer, and incubated for 10 min on ice. The incubation was followed by centrifugation at RT for 10 min at 4500 x g. The supernatant was transferred into a fresh falcon tube, mixed with 4.8 mL of 95% ethanol, and the samples were incubated for 10 min at RT. The samples were centrifuged at RT for 10 min at 4500 x g, the supernatant was discarded, and the obtained pellet was incubated with 1 mL of 70% ethanol for 1 min at RT. The ethanol was removed, and the pellet was mixed with 500 µL of Tris-EDTA buffer by vortexing for 30 s. The samples were incubated at 50 °C in a water bath for 1 h, vortexed, and the DNA was transferred into a fresh tube.

The DNA concentration was determined using Qubit dsDNA HS (high sensitivity, 0.2 to 100 ng) Assay Kit and Qubit 3.0 fluorometer (Life Technologies) according to the manufacturer’s protocol. The reference samples, including 0 and 10 ng of DNA, were provided with the kit. A sample (reference samples and extracted DNA samples) volume of 1 µL was mixed with 199 µL of a Qubit working solution, incubated for two minutes, briefly centrifuged, and measured with Qubit 3.0 fluorometer (Life Technologies). The samples were diluted to achieve 500 ng of DNA at a concentration of ~ 10 ng/ µL and were stored at – 80 °C until the sequencing.

### Genome sequencing

500ng of genomic DNA was sheared to 200-700 bp size distribution by Adaptive Focused Acoustics using a Covaris E220 instrument (Covaris, Inc.) under the following conditions: 50 ul total volume, 10% duty cycle, 175 intensity, 200 cycles per burst, 50 s in frequency sweeping mode. The remainder of the library preparation followed the manufacturer’s protocol described in the NEBNext Ultra II DNA Library Prep kit for Illumina (Catalog No. E7645S, New England Biolabs, Inc.). Briefly, the sheared DNA was end-repaired and A-tailed to generate blunt ends, then ligated to Illumina-compatible adaptors, followed by size selection using Agencourt AMPure XP Beads (Beckman Coulter, Inc.). Finally, the adaptor-ligated DNA fragments were PCR enriched [10]. The quality of the final NGS libraries was assessed using an Agilent Bioanalyzer high-sensitivity chip (Agilent Technologies, Inc.). Each sample showed a narrow distribution with a peak size of approximately 300 bp. Each sample was sequenced in two lanes on the Illumina HiSeq 4000 in a paired-end 150 bp run (300 cycles).

### Variant calling

Raw reads were aligned to the reference genome Hg19 using BWA-MEM algorithm [11]. Downstream processing was carried out with the Genome Analysis Tool Kit (GATK) best practice [12], Samtools [13], and Picard tools (http://broadinstitute.github.io/picard). Variants were called using GATK HaplotypeCaller. All calls with a Phred-scaled quality ≤ 30 were filtered out [14]. Mean coverage was ~ 30X for all samples.

### Causal variant identification

The resulting VCF files were uploaded to Ingenuity Variant Analysis (IVA, Qiagen) and processed using the following filter cascade (Figure S1): (1) exonic region filtering, leaving 53,696 variants in 14,437 genes; (2) variant call confidence filtering (call quality > 20 and outside of the top 5% most exonically variable 100base windows in healthy public genomes), leaving 35,158 variants in 12,294 genes; (3) common variant filtering (exclude all variants with allele frequency > 0.5% in the 1000G, ExAC, gnomAD, and NHLBI ESP exomes databases), leaving 9,869 variants in 4,007 genes; (4) deleterious variant filtering (keep only experimentally observed pathogenic or likely pathogenic variants, or frameshift, in-frame indel, start/stop codon changes, or splice site loss variants) leaving 7,323 variants in 2,996 genes; (5) genetic recessive inheritance filtering (homozygous for the alternative allele in both affected samples, heterozygous in both parents, either heterozygous or reference allele in all siblings), leaving four variants (Table S1). Three were missense variants of uncertain significance (chr8:87060924 in *PSKH2* (*p.Met309Leu*, transcript ID *= ENST00000276616*), chr8:110657485 in *SYBU* and *LOC100132813* (*p.Pro79Arg*, transcript ID *= ENST00000276646*), chr10:88466383 in *LDB3* (*p.Ala331Val*, transcript ID *= ENST00000361373*)) and one was a stop gain variant (chr8:96259914 in *C8orf37* (p.Trp185*, transcript ID *= ENSP00000286688*)). The characteristics [15–17] of the candidate variants are summarized in Table S1. The three missense variants have not been reported in association with any disease before, while the stop gain variant in *C8orf37* (rs748014296) has been confirmed in patients with retinitis pigmentosa [18]. The stop gain variant in *C8orf37 -* classified as pathogenic by the American College of Medical Genetics and Genomics *-* was considered the causal variant and no further analysis of structural variants was conducted.

### Variant confirmation

The genotype of the variant in *C8orf37* (p.Trp185*) of the two affected individuals was confirmed by a CLIA-certified lab for molecular genetic diagnostic testing of whole blood collected on filter paper (Centocard) and shipped to Centogene labs (Centogene, Germany). Targeted sequencing was performed at Centogene on both DNA strands of the relevant *C8orf37* region. The reference sequence used was NM_177965.3, and the variant in *C8orf37* (p.Trp185*) was confirmed in homozygous form in both affected samples.

### Corneal confocal microscopy

CCM (Heidelberg Retinal Tomograph III Rostock Cornea Module; Heidelberg Engineering GmbH) was carried out on the family. The examination took approximately 10 min for both eyes, and a single experienced examiner (AK), masked from the patient’s condition, performed CCM and acquired images using the “section” mode. Based on depth, contrast, and focus, six images per subject (three per eye) were selected [19] and analyzed from the central sub-basal nerve plexus. Images were analyzed using validated, purpose-written software (CCMetrics; M. A. Dabbah, ISBE, University of Manchester, Manchester, UK). Corneal nerve branch density (CNBD), corneal nerve fiber density (CNFD), and length (CNFL) were quantified according to a previously established protocol [9]. Data was averaged for the left and right eyes for each patient.

### Optical coherence tomography

Spectral-domain optical coherence tomography (SD-OCT) (Spectralis OCT, Heidelberg Engineering GmbH, Heidelberg, Germany) was performed. Participants were asked to fixate on an internal fixation light to perform the scan. The RNFL measurement was performed by manually positioning a scan circle of approximately 3.45 mm (scanning angle = 12 degrees) at the center of the optic disc. RNFL measurement was performed 3 times per patient. The built-in software generated a color-coded significance map, and average RNFL values were automatically calculated for Nasal (N) / Nasal-Superior (NS) / Nasal-Inferior (NI), Temporal (T) / Temporal-Superior (TS) / Temporal-Inferior (TI) and Global (G) regions of the optic nerve head.

## Results

Whole genome sequencing was performed using DNA from saliva obtained from the two patients, four unaffected siblings, and the parents who were first-degree cousins. A previously reported pathogenic stop gain variant in *C8orf37* (p.Trp185*, rs748014296), known to cause BBS type 21, was found in the homozygous state in both affected individuals. The variant was confirmed in blood from both patients by independent genetic diagnostic testing in a certified laboratory.

**Fig. 2:**
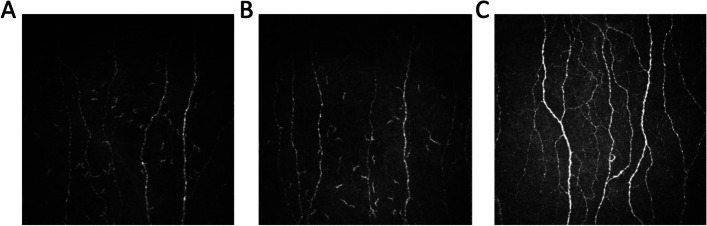
Corneal nerve images of (**A**) the affected 31-year-old male (proband) and (**B**) the affected 33-year-old female (affected sibling) showing a loss of corneal nerve fibres compared to (**C**) the healthy male sibling

**Fig. 3:**
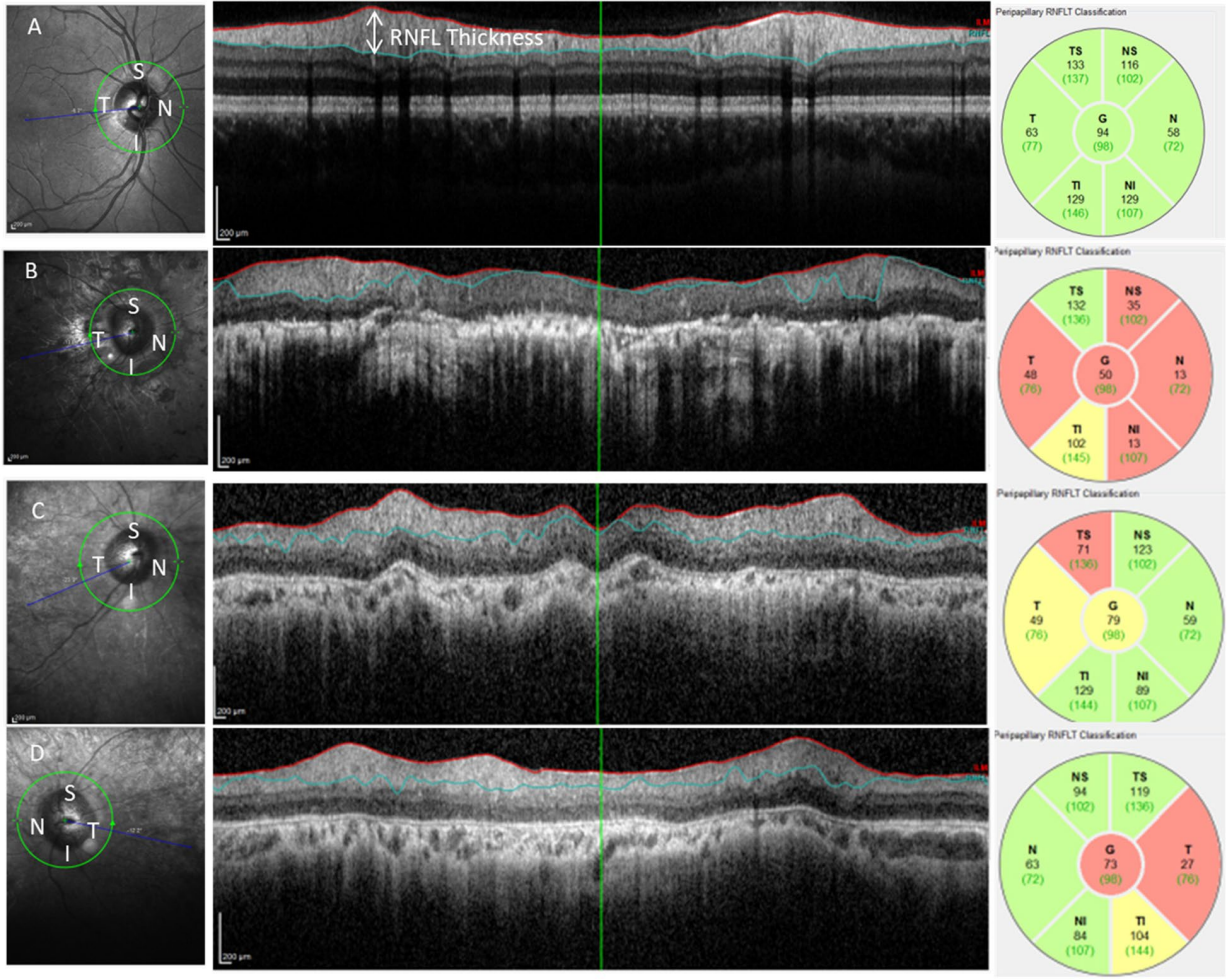
**A** RNFL measurements of the right eye of a control subject; (**B**) the right eye of the proband with thinning of the RNFL in all quadrants except the superior temporal region; (**C**) RNFL measurements of the right eye of the affected sibling showing thinning of the RNFL in the superior temporal quadrant; (**D**) RNFL measurements of the left eye of the affected sibling showing thinning of the RNFL in the temporal quadrants

Both patients had a lower CNFD, CNBD and CNFL compared to their unaffected sibling (Fig. 2; Table 1). The proband had pendular nystagmus and retinal examination revealed retinitis pigmentosa with waxy disc pallor, arteriolar attenuation, peripapillary atrophy, generalized macular-dystrophy and bone-spicule like pigmentation of the peripheral retina (Fig. 3B). Compared to the normative range (control Fig. 3A) there was a marked reduction in the thickness of the RNFL in the Global, Nasal, Superior Nasal, and Inferior Nasal regions, with moderate RNFL thinning in the Temporal and Inferior Temporal regions and sparing of the Temporal Superior region (Fig. 3B; Table 1). It was not possible to scan the left eye due to pendular nystagmus. The affected sibling also had macular dystrophy (Fig. 3C). OCT of the right eye showed a reduction in the thickness of the RNFL in the Global, Temporal and Superior Temporal regions, while the other regions were normal (Fig. 3C; Table 1). The left eye also showed a reduction in the thickness of the RNFL in the Global, Temporal and Inferior Temporal regions, while the other regions were normal (Fig. 3D; Table 1).

**Table 1:** Clinical characteristics and results of corneal confocal microscopy and optical coherence tomography in the affected individuals and their healthy sibling. ^*^Normal laboratory range for cholesterol, triglyceride, HDL, LDL and HbA1c provided

Parameters	Proband		Affected sibling		Healthy sibling	
Age (years)	32		33		26	
Gender (male/female)	Male		Female		Male	
Cholesterol, mmol/l	4.7		4.0		<5.2^*^	
Triglycerides, mmol/l	2.0		1.3		<1.7^*^	
HDL, mmol/l	0.8		1.3		>1.6^*^	
LDL, mmol/l	3.0		2.2		<2.6^*^	
HbA1c (%)	8.2		5.5		<5.7^*^	
**Corneal Confocal Microscopy**						
CNFD, no./mm^2^	29.2 ± 9.3		31.3 ± 10.8		40.6 ± 8.7	
CNBD, no./mm^2^	22.9 ± 13.3		43.8 ± 32.9		73.9 ± 24.9	
CNFL, mm/ mm^2^	14.8 ± 3.3		16.6 ± 5.9		22.9 ± 4.6	
**Optical Coherence Tomography**						
Parameters	Proband		Affected sibling		**Control**	
Eye	Right	Left	Right	Left	Right	Left
Nasal Superior, (µm)	35		123	94	116	123
Nasal, (µm)	13		50	63	58	50
Nasal Inferior, (µm)	13		89	64	129	143
Temporal Inferior, (µm)	102		129	104	129	125
Temporal, (µm)	48		49	27	63	66
Global, (µm)	50		79	73	94	96
Temporal Superior, (µm)	132		71	119	133	147

## Discussion

We used whole genome sequencing to identify a *C8orf37* stop-gain variant in a patient presenting to the endocrine clinic with obesity and diabetes with syndromic features. The *C8orf37* stop-gain variant identified here (p.Trp185*) was previously reported to cause BBS in a consanguineous Pakistani family with an identical clinical phenotype [18]. A variant in *C8orf37* has been associated with autosomal-recessive retinal dystrophies and macular involvement [20, 21].

A key feature of BBS is vision loss due to a rod-cone dystrophy, affecting the outer retina which may lead to inner retinal diminution [1, 3]. Consistent with previous reports [22, 23], both patients had retinitis pigmentosa and night blindness occurring in the first decade of life, reaching legal blindness between the 2nd and 3rd decade of life. One may assume that inner retinal structures such as the retinal nerve fibre layer (RNFL) will be preserved in patients with outer retinal disease. However, clinically evident RNFL thinning has been reported even on fundus photography in various diseases of the outer retina, including Best macular dystrophy, Leber congenital amaurosis, Stargardt disease, choroideremia and rod-cone dystrophy [24]. And yet, two previous reports using OCT showed no change in the RNFL of patients with BBS [5, 25]. Studies have shown that Fourier-domain OCT may detect peripapillary RNFL thinning in patients with retinitis pigmentosa [26, 27]. We have used state of the art spectral-domain optical coherence tomography (SD-OCT), and indeed find a patchy thinning of the RNFL in both patients, suggestive of a retinal abnormality extending beyond rod-cone dystrophy in patients with BBS.

In addition to vision loss, anosmia, and defective hearing have also been described in patients with BBS, indicating a more widespread defect in sensory perception [1]. Indeed, in experimental studies of *Bbs1*^−/−^ and *Bbs4*^−/−^ knockout mice there was evidence of altered thermal and mechanical perception after excluding motor and higher cortical dysfunction, with hyperinnervation of dermal papillae and preserved intraepidermal nerve fibre endings [7]. We have used CCM, a rapid, reproducible ophthalmic technique that shows corneal nerve fiber loss which relates to altered thermal perception and loss of intraepidermal nerve fibres in diabetic neuropathy [28, 29] and several hereditary neuropathies including Charcot-Marie-Tooth disease type 1 A (CMT1A) [30] and Friedreich ataxia [31]. We show a marked loss of corneal nerve fibers in both affected siblings, consistent with severe small fibre neuropathy.

In summary, we report the clinical utility of rapid genomic sequencing from saliva to identify a *C8orf37* variant, enabling the diagnosis of BBS in a patient with a common presentation of obesity and diabetes but with unusual additional clinical features. We also highlight the utility of ophthalmic technologies to identify novel pathological changes in the retina and corneal nerve fibres of patients with BBS, extending the clinical phenotype of these patients. Corneal confocal microscopy in particular enabled the identification of neurodegeneration in BBS, which could be a primary feature of BBS, secondary to diabetes or a combination of both.

### Supplementary Information

**Additional file 1: Table S1. **Characteris1cs of the variant candidates.** Figure S1. **The workflow for discovering the disease-causing variant for the BBS family.

## Data Availability

Raw genomics data cannot be shared publicly based on the informed consent given by the participants. However, data can be made available upon request from the corresponding author subject to approval by the Weill Cornell Medicine Institutional Data Access / Ethics Committee following institutional policies.

## Abbreviations

BBS: Bardet-Biedl syndrome
OCT: Optical coherence tomography
T2DM: Type 2 diabetes mellitus
CCM: Corneal confocal microscopy
RT: Room temperature
GATK: Genome Analysis Tool Kit
CNBD: Corneal nerve branch density
CNFD: Corneal nerve fiber density
CNFL: Corneal nerve fiber length
SD-OCT: Spectral domain optical coherence tomography
RNFL: Retinal nerve fiber layer
