# Supplementary material for: Retinal nerve fibre layer thinning and corneal nerve loss in patients with Bardet-Biedl syndrome
Source: BMC Med Genomics. 2023 Nov 23;16:301. doi: 10.1186/s12920-023-01739-w (PMC10666305; doi:10.1186/s12920-023-01739-w)
Supplement: Supplementary file 1 — Additional file 1: Table S1. Characteris1cs of the variant candidates. Figure S1. The workflow for discovering the disease-causing variant for the BBS family. [file 12920_2023_1739_MOESM1_ESM.pdf]

Table S1. Characteristics of the variant candidates.

Gene	Variant	Position	Consequence	Type	Gnomad MAF	GERP prediction	CADD score
PSKH2	rs768373520	chr8:87060924	p.Met309Leu	Missense	3.98e-06	2.24 (constrained)	15 (likely benign)
C8orf37	rs748014296	chr8:96259914	p.Trp185*	Stop gain	7.96e-06	1.93 (likely constrained)	43 (deleterious)
LDB3	rs368053281	chr10:88466383	p.Ala331Val	Missense	5.69e-05	3.46 (constrained)	7 (likely benign)
LOC100132813 & SYBU	rs1343269348	chr8:110657485	p.Pro79Arg	Missense	3.69e-05	0.06 (likely unconstrained)	7.225 (likely benign)

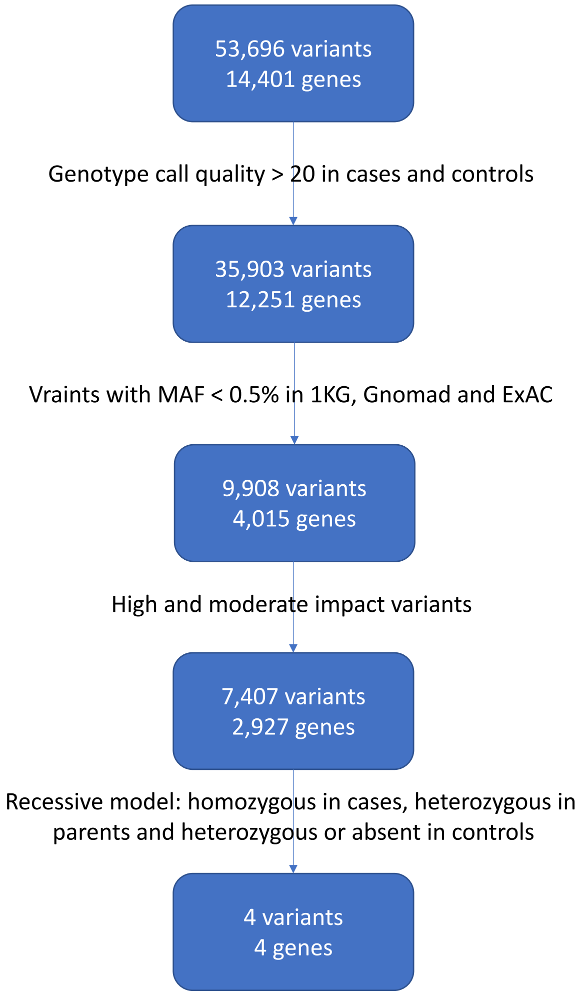

Figure S1. The workflow for discovering the disease-causing variant for the BBS family. The disease-causing variant was identified by filtering steps including high genotype call quality, low frequency in public databases, falling within coding/splicing regions, pathogenicity analysis and recessive mode of inheritance.
